# Cross‐Analysis of Single‐Cell Transcriptomic Datasets Reveals Conserved Neurogenic Gene Signatures and New Insights Into Neural Stem Cell Aging

**DOI:** 10.1111/acel.70106

**Published:** 2025-06-04

**Authors:** Oliver Polzer, E. Kinloch, P. J. Lucassen, E. Salta, C. P. Fitzsimons

**Affiliations:** ^1^ Brain Plasticity Group, Swammerdam Institute for Life Sciences University of Amsterdam Amsterdam the Netherlands; ^2^ Laboratory of Neurogenesis and Neurodegeneration Netherlands Institute for Neuroscience Amsterdam the Netherlands

**Keywords:** adult hippocampal neurogenesis, neural stem cells, quiescence, senescence, single cell transcriptomics

## Abstract

Hippocampal adult neural stem cells (NSCs) contribute to neurogenesis and astrogliogenesis throughout life. They play multifaceted roles in hippocampal function, including memory processing, stress regulation, and cognitive flexibility. Located in unique neurogenic niches like the subgranular zone of the hippocampal dentate gyrus, NSCs exhibit notable heterogeneity and can be classified into quiescent, activated, and intermediate transitioning states. This diversity, while instrumental to their adaptability and function, presents challenges in molecular classification and functional interrogation. Here, we discuss current limitations and compare NSC transcriptional profiles from publicly available single‐cell RNA sequencing datasets. We address discrepancies in NSC classification between studies, identify conserved gene expression profiles, and propose new markers that could serve as standardized references. Furthermore, we explore how pseudotime inference analyses provide insights into the temporal dynamics of NSCs and their progression toward neural progenitors, further aiming to optimize their classification. We also examine cellular changes in NSCs during aging and explore the potential of these cells to undergo senescence. Our work helps to resolve inconsistencies in current cell‐type annotations in literature and proposes a framework to study and classify the different states of NSCs, thereby offering a better understanding of their dynamic roles in neurogenesis, aging, and cellular senescence.

## Graphical abstract

Here, we reanalyze single‐cell RNA sequencing datasets demonstrating hippocampal adult neural stem cell (NSC) heterogeneity to resolve classification inconsistencies and identify conserved gene markers. Our findings enhance understanding of NSC dynamics, aging, and senescence, providing a framework for standardized classification and improved insights into their roles in neurogenesis and cognitive function.

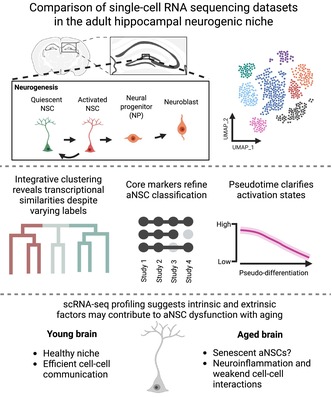

## 1 Introduction

Adult neural stem cells (NSCs) give rise to neurons and glial cells throughout life in multiple mammalian species (Taupin and Gage 2002; Moreno‐Jiménez et al. 2021). During development, neuro‐ and astroglio‐genesis, starting from embryonic NSCs, shape the brain and populate it with diverse cell types. However, in the adult brain, NSCs are limited to permissive, highly regulated local microenvironments, termed “neurogenic niches” (Bond et al. 2015). Well‐documented neurogenic niches in rodents include the subventricular zone (SVZ) lining the lateral ventricles and the subgranular zone (SGZ) of the dentate gyrus (DG) in the hippocampus (Alvarez‐Buylla and García‐Verdugo 2002; Kempermann et al. 2015; Llorente et al. 2022).

In the DG, new neurons that originate from NSCs play a significant role in hippocampus‐dependent memory functions, including pattern separation and cognitive flexibility, as well as in regulating behavioral and physiological responses to stress (Anacker and Hen 2017; Surget and Belzung 2022). Furthermore, NSCs can give rise to adult‐born astrocytes, which may support synaptic activity and provide metabolic support to neurons (Schneider et al. 2022). Interestingly, NSCs can also exhibit autocrine and paracrine functions linked to their capacity to generate new cells. For example, Sox2‐dependent secretion of growth factors, sonic hedgehog (Shh), Milk Fat Globule‐EGF 8 (Mfge8) or the stem cell‐derived neural stem/progenitor cell supporting factor has been identified as important NSC‐secreted signals influencing their own or adjacent cellular dynamics within the neurogenic niche microenvironment (Toda et al. 2003; Favaro et al. 2009; Zhou et al. 2018). Remarkably, initial results indicate that NSCs in pathological conditions, such as epilepsy and traumatic brain injury, can develop functional features shared by other glial cells, such as the ability to phagocytose or contribute immunomodulatory functions linked to the pathological hyperactivity in the DG (Abiega et al. 2024). These and similar observations have suggested that NSC preservation and “rejuvenation” may be suitable strategies for the treatment of aging‐associated diseases that affect hippocampal function, resulting in cognitive decline (Negredo et al. 2020).

Previous studies have shown that NSCs are comprised of heterogeneous cell populations, even within the local environment of their specific neurogenic niches (DeCarolis et al. 2013). Therefore, a better understanding of the diversity of NSCs and the differences between them is crucial for employing or targeting NSCs for treating aging‐associated diseases. While the diversity of NSC populations was initially characterized solely based on molecular profiling, it is now clear they also represent different functional groups (Bottes et al. 2021; Pilz et al. 2018). Some of these populations remain largely quiescent, while others get activated and give rise to new cells. After activation, they can return to quiescence or become depleted from the existing NSC pool (Pilz et al. 2018; Bonaguidi et al. 2011; Götz 2018). So far, an accurate characterization of the different populations that comprise this heterogeneous NSC pool has proven to be a complex task.

Several techniques and experimental systems have been employed to characterize NSC populations, including inducible transgenic mouse lines expressing fluorescent proteins under the control of specific promoters. In addition, the recent development of single‐cell and single‐nuclei RNA sequencing techniques (scRNA‐seq/snRNA‐seq) has provided powerful experimental tools to identify, profile, and classify new cell types, including rare NSC populations (Tosoni et al. 2023).

To that end, we here focus on NSCs in the mouse hippocampal SGZ, review their characteristics, and compare and analyze publicly available scRNA‐seq datasets describing their transcriptional profiling. We address current limitations in mapping NSC heterogeneity and discuss the insights that can be derived from the use of scRNA‐seq technologies. By cross‐analyzing several previously published datasets, we identify sets of conserved genes across studies, which we suggest can be used as advanced markers to study NSC populations. Finally, we propose that these markers can be used to identify the different states NSCs may undergo throughout their lifespan. They may also allow us to unveil how NSCs can be affected by aging and transition into cellular senescence. Overall, our review of the literature and cross‐analysis of published datasets provides a useful resource to neuroscientists and cell biologists interested in understanding NSC behavior and its functional implications for the aging hippocampus.

### 1.1 The Multiple Dynamic Fates of NSCs

Throughout adulthood, most NSCs in the SGZ are found in quiescence, that is, a reversible cell cycle arrest in the G_0_ phase of the cell cycle (Urbán et al. 2019). Traditionally, the annotation NSC is reserved for multipotent cells able to proliferate, self‐renew and produce progeny, that will differentiate into neurons, astrocytes and oligodendrocytes and the annotation “neural progenitor (NP) cell” applies to the non‐stem cells progeny that has the capacity to proliferate and differentiate, but limited self‐renewal capacity (Eckert et al. 2017). Although not fully demonstrated for NSCs yet, data from other somatic stem cells indicate that quiescence may be crucial to protect long‐lived stem cells from the accumulation of replication‐associated DNA damage. However, this protective mechanism may come at the cost of lower expression of DNA repair factors and increased vulnerability when stem cells exit quiescence at later age (Tümpel and Rudolph 2019). Furthermore, the maintenance of NSC quiescence not only requires energy, but also a complex molecular program to suppress terminal differentiation and a drift to irreversible, non‐proliferative states (Marescal and Cheeseman 2020; Urbán and Cheung 2021; Scandella et al. 2023). This tightly regulated dynamic equilibrium can be disturbed by several stimuli, inducing an exit from quiescence and leading to NSC “activation.” NSC activation promotes the transition to other cellular states, such as proliferation, fate specification and differentiation (Urbán and Cheung 2021). In many cases, exit from quiescence is triggered by mitogenic signals that promote the expression of cell‐cycle proteins, such as cyclin dependent kinases (Laurenti et al. 2015).

To further understand NSC behavior following activation, studies have explored their activation dynamics and self‐renewal properties. A “disposable stem cell” model has been proposed, based on nucleotide analogue labeling in a Nestin‐GFP reporter line, indicating rapid rounds of asymmetric divisions, leading to a depletion of the NSC pool (Encinas et al. 2011). In line with this model, other studies using intravital imaging and clonal analysis with specific gene promoters that label only specific subsets of NSCs, such as Ascl1‐CreER^T2^‐GFP (Bottes et al. 2021; Pilz et al. 2018; Ibrayeva et al. 2021), Gli1‐CreER^T2^‐GFP (Bottes et al. 2021; Wu et al. 2023), or Nestin‐CreER^T2^‐GFP (Ibrayeva et al. 2021) suggest that in particular Ascl1‐positive NSC populations represent short‐lived NSCs, that are rapidly depleted after several cycles of division. Interestingly, other NSC populations labeled by the activity of the Gli1 or Nestin promoters, may retain self‐renewal capability over time, and may act as long‐term NSCs (Encinas et al. 2011; Ibrayeva et al. 2021), providing an example of functional heterogeneity of NSCs. Recent observations using thymidine analogue 5‐ethynyl‐2′‐deoxyuridine (EdU) labeling combined with immunostainings, rather than using specific reporter lines (Harris et al. 2021), suggest that NSCs in young mice are depleted fast, in line with the disposable stem cell model (Encinas et al. 2011); however, these dynamics change with aging. Some NSCs populations (termed resting NSCs) return to quiescence after activation, while other populations (termed dormant NSCs), remain quiescent or have not been activated before as evidenced by EdU injections combined with Ki67 labeling (Harris et al. 2021).

The process of NSCs activation and proliferation in the SGZ is highly complex and involves various levels of gene regulation, ranging from transcription, RNA processing, noncoding RNA‐mediated mechanisms, and protein synthesis to shifts in metabolism (for a more detailed review, see Urbán et al. (2019); Penning et al. (2021); Olpe & Jessberger (2023)). As mentioned before, several studies have used transgenic mouse lines that label only specific subsets of NSCs, which might have obscured a more complete view of the whole NSC pool (Olpe and Jessberger 2023). Indeed, the commonly used Glast‐CreER^T2^ and Nestin‐CreER^T2^ transgenes might label distinct NSC populations, highlighting their heterogeneity (DeCarolis et al. 2013). Therefore, future work should move away from traditional approaches that rely on single promoters that do not label the complete NSC pool, toward experimental approaches that require co‐expression of two or more distinct markers.

This experimental shift could provide more extensive and precise labeling, helping to uncover the full diversity within the NSC pool. One such method is the Split‐Cre system (Beckervordersandforth et al. 2010; Hirrlinger et al. 2009), which could be used in studies involving a range of different ages. Indeed, a Split‐Cre approach in which the C‐terminal half of the Cre recombinase is driven by the Prominin1 P2 promoter and inserted into one lentiviral vector, while the N‐terminal half of Cre is driven by hGFAP promoter elements and inserted into a second lentiviral vector has been used to target NSC in the DG in vivo (Beckervordersandforth et al. 2010; Pons‐Espinal et al. 2017; Schouten et al. 2019). This and other combinatorial expression approaches with the use of a standardized set of marker genes could be used to address the outstanding question of whether this heterogeneity is dictated by specific subpopulations, or if the entire pool of NSCs can potentially acquire the full spectrum of activation states (Beckervordersandforth et al. 2010).

### 1.2 Challenges in Classifying the Heterogeneity of NSCs: Marker Choice, Activation States, and Morphological Diversity

As discussed in the previous section, the inherent dynamics between NSC populations represent a major challenge when characterizing the heterogeneity of NSCs in the SGZ. Several technical and conceptual limitations also influence NSC classification, including marker choice, defining activation states, and morphological diversity. In this section, we will discuss this problem in more detail.

The conventional characterization of NSC populations has so far relied on “cell type‐specific” markers, often using specific antibodies like Sox2, GFAP, and/or Nestin (Table 1). Nestin is an intermediate filament protein highly expressed in NSCs and other cell types of the SGZ, such as NPs, astrocytes, and cells with an oligodendrocyte signature (Tosoni et al. 2023; Artegiani et al. 2017; Walgrave et al. 2021; Bielefeld et al. 2024). Sox2 is a transcription factor essential for maintaining NSCs, but it is also widely expressed in astrocytes. GFAP, a common astrocyte marker, is also expressed in NSCs and early progenitor cells. These three markers are frequently used in combination along with morphological features to include or exclude specific populations (Kempermann et al. 2004).

**TABLE 1:** List of studies identifying neural stem cell populations.

Name of NSC	Animal model								Markers											Functional state markers											References
																				Proliferation					Senescence						
	F344 rats	C57BL6	Hes5: GFP	Nestin‐GFP	GFAP‐GFP	Gli1	LPA1‐GFP	Ascl1	Sox2	GFAP	Nestin	BLBP	HopX	Vimentin	S100b	Sox1	Prominin1	GLT1	LPA1	Ki67	BrdU	EdU	Mcm2	PCNA	SA‐β‐Gal	p16	p15	Il6	γH2AX	LamininB	
Astrocytes/NSCs	X								+	+																					Hattiangady, Shetty (2008)
Radial glia	X								+					+																	Hattiangady, Shetty (2008)
Sox2 immunoreactive cells	X								+											+	+										Hattiangady, Shetty (2008)
Radial glia like cells (R cells)		X							+	+			+		−																Cole et al. (2022)
Radial NSCs			X						82%	66%		74%									+			+							Lugert et al. (2010)
Horizontal NSCs			X						+	+		+									+			+							Lugert et al. (2010)
Radial glia like cells (RGLs)				X					+	+	+																				Bonaguidi et al. (2011)
Non‐radial precursors				X					+																						Bonaguidi et al. (2011)
QNPS				X		X			+	+	+	+		+							+										Encinas et al. (2011)
ANPS				X		X			+		+	+		−							+										Encinas et al. (2011)
Astrocyte progenitors				X		X			+	+	−	+			−																Encinas et al. (2011)
Alpha‐cells				X	X				+	+	+				−	+	+	−		+	+										Gebara et al. (2016)
Beta‐cells				X	X				+	+	18%				+	49%	32%	+		+	+										Gebara et al. (2016)
Alpha‐cells				X					+	+	GFP+	+			−				+		+										Martín‐Suárez et al. (2019)
Beta‐cells				X						+	GFP+				+						+										Martín‐Suárez et al. (2019)
Omega‐cells				X					+	+	GFP+	+			−				+		+										Martín‐Suárez et al. (2019)
Dormant NSCs		X							+	+					−						−	−	+								Harris et al. (2021)
Resting NSCs		X							+	+					−						−	+	+								Harris et al. (2021)
Proliferating NSCs		X							+	+					−						+	+	+								Harris et al. (2021)
Short‐term NSCs								X		+													+								Ibrayeva et al. (2021)
Long‐term NSCs				X						+	+												+								Ibrayeva et al. (2021)
Radial NSCs						X			+		+									+											Bottes et al. (2021)
Radial NSCs								X	+		+									+											Bottes et al. (2021)
Non‐radial percursors						X		X	+		−									+											Bottes et al. (2021)
R cells				X																+											Wu et al. (2023)
NR cells				X																+											Wu et al. (2023)
R cells						X			+	+					−					+											Wu et al. (2023)
NR cells						X														+											Wu et al. (2023)
Senescent NPCs		X							+	+										+	+				+	+	+	+	+	+	Fatt et al. (2022)
R cells							X		+	+	GFP+				−					+											Walker et al. (2016)
NR cells							X		+	+	GFP+									+											Walker et al. (2016)

*Note:* The list shows the populations identified with the original name used in the study, along with the mouse strains and immunohistochemistry markers used for identification. The numbers represent the percentage of cells found to be positive within the total population. The + and − signs indicate the presence or absence of specific markers. GFP indicates that the marker is derived from the transgenic mouse model used.

The difficulty of marker choice increases when specific activation states of NSCs are considered in the analysis. Ki67 and 5‐bromo‐2′‐deoxyuridine (BrdU) are commonly used to define proliferative states (Kee et al. 2002). Ki67, encoded by the *Mki67* gene, is a protein expressed during the G_1_, S, G_2_, and mitosis phases of the cell cycle but is absent from G_0_, commonly used as a proliferation marker (Cuylen et al. 2016). BrdU, on the other hand, is a synthetic nucleoside analogue that labels actively dividing cells by incorporation into DNA during the S phase, marking DNA replication. In contrast, other studies have relied on the expression of cell cycle proteins, such as cyclin‐dependent kinases and other activation markers, which are expressed at higher levels during specific cell cycle phases that reflect a broader range of cell cycle activity rather than just the DNA synthesis phase. Therefore, if proliferation is defined as completed cell division (Schafer 1998), then it can be conceptualized as a cell state different from activation.

Extending the complexity of NSC cell state definitions, the state of quiescence, characterized by having exited the cell cycle and entered a reversible G_0_ phase state (Cheung and Rando 2013), so far lacks a specific marker for immunolabeling and often relies on the absence of proliferation markers, or genetic modification in transgenic mouse lines (Oki et al. 2014). Interestingly, scRNA‐seq studies support Aldoc and/or Hopx as potential markers for quiescent NSCs (qNSCs), as Aldoc marks NSCs that do not express the proliferation marker PCNA, and Hopx is highly expressed in qNSCs and downregulated during their transition to activated NSCs (aNSCs) (Shin et al. 2015). However, other more recent scRNA‐seq studies have shown that Aldoc and Hopx are also expressed in astrocyte populations derived from NSCs (Bielefeld et al. 2024; Karpf et al. 2022).

Based on morphology, NSCs can further be separated into radial glia‐like (RGL) and non‐radial glia‐like (NR) cells. RGL cells are largely quiescent with a characteristic elongated shape and extended processes, while NR cells are more rapidly cycling, displaying a more rounded shape with shorter processes. However, not all RGL‐NSCs are uniformly positive for Sox2, GFAP, or Blbp (Lugert et al. 2010). The complexity of this approach to identify RGL cells using immunohistochemical markers and morphological features further exemplifies the difficulty in distinguishing them from similar but different cell types, such as astrocytes.

Further advancing our understanding of NSC morphological diversity, Gebara et al. (2016) characterized the morphology of RGL cells with various markers in a GFAP‐GFP and a Nestin‐GFP mouse line, generating a new NSC (sub)classification (Gebara et al. 2016). They defined two distinct cell populations, so‐called morphophenotypes, whose relative proportions change with age: (1) Type α RGL cells, constituting about three‐quarters of the NSC pool in 8‐week‐old mice. Type α RGL cells display a long primary process that modestly branches into the molecular layer. Type α RGL cells can be activated, giving rise to neurons, astrocytes, and type β RGL cells. (2) Type β RGL cells, comprising the remaining one quarter of the RGL cells in younger mice, display a shorter radial process with highly branching extensions into the outer granule cell layer‐inner molecular layer border. Importantly, type β RGL cells also express markers of mature astrocytes, such as Glt1 and S100ꞵ. Type β RGL cells predominate in the older brain, do not proliferate, and spontaneously transform into astrocytes, defined by their typical stellar morphology and immunohistology against GFAP (Gebara et al. 2016).

These observations raise the question of whether RGL cells remain deeply quiescent, become senescent or undergo a programmed transition into astrocytes. Importantly, type β RGL cells in the DG of old mice can be activated and proliferate following glucocorticoid receptor knockdown (Schouten et al. 2019), indicating that they retain proliferative capacity. Regarding marker heterogeneity, although both populations express Sox2 and GFAP, only 18% of the type β cells were positive for Nestin (Gebara et al. 2016). A later study described a third RGL cell morphophenotype, namely type Ω RGL cells. Despite sharing morphological similarities with type β RGL cells and being present in the aged brain, type Ω RGL cells are less likely to divide even under pro‐activation conditions. Unlike type ꞵ RGL cells, they lack the expression of S100ꞵ, but do share the expression of Nestin‐GFP and GFAP (Martín‐Suárez et al. 2019).

Overall, these findings suggest that using markers like Nestin or others as universal NSC identifiers can be problematic; distinctions between NSC subtypes are not straightforward, even when combining multiple markers with morphological characteristics. Highlighting this complexity, a reanalysis of two scRNA‐seq datasets aimed to determine a molecular signature of mouse RGL cells in the DG has suggested that putatively rare RGL populations could be “masked” by co‐clustering astrocytes, particularly in smaller datasets (Tosoni et al. 2023).

The difficulty of characterizing NSCs extends beyond molecular markers alone to the inclusion of morphological features, as well as the consideration of different activation states and their similarities to astrocytes. Moreover, discrepancies in nomenclature, along with the use of various transgenic reporter lines and the classification methodologies across studies (Table 1), further complicate our understanding of the different NSC subpopulations. Addressing these challenges is essential for advancing our knowledge of NSC heterogeneity and for facilitating meaningful comparisons across studies.

Along these lines, a unifying nomenclature of NSCs is essential. This should include consideration of marker choice, mouse strain, age, and other experimental conditions when defining NSC populations across studies. By doing so, we could improve the systematic classification and understanding of NSC heterogeneity, as all these factors may differentially influence NSC populations. Importantly, (re‐)analysis of scRNA‐seq datasets has shown a direct comparison of NSC populations, based on their transcriptomic profile obtained from independent, but technically dissimilar scRNA‐seq experiments, is possible (Tosoni et al. 2023; Bielefeld et al. 2024).

In the following sections, we show how comparing different publicly available scRNA‐seq datasets could help overcome some of these current limitations and facilitate a more holistic understanding and classification of NSC populations in the DG and of their properties.

## 2 Results and Discussion

### 2.1 Decoding NSC Complexity One Cell at a Time: Lessons and Challenges From scRNA‐Seq Studies

In recent years, scRNA‐seq has become a powerful tool to elucidate genome‐wide transcriptomic changes, allowing the profiling of individual cell populations, including rare cell populations in complex tissues. Yet, the identification of NSCs, especially in humans, remains a substantial challenge, and various experimental and biological factors have hindered a complete understanding (Tosoni et al. 2023). ScRNA‐seq datasets offer the possibility to compare the transcriptional profiles of NSCs between different studies, which could yield more insight into the dynamics and reveal new characteristics or markers of diverging NSC populations and states, contributing relevant information for their (sub)type classification.

### 2.2 Comparative Analysis and Gene Marker Identification Using Public‐Available scRNA‐Seq Datasets

To dissect commonalities and differences among identified NSCs, NP and neuroblast cell clusters, we compared cellular populations from seven recent scRNA‐seq studies focused on the hippocampus. These studies were selected due to the availability of freely accessible data and the presence of all necessary metadata for analysis. To facilitate the assessment of the comparability of the datasets and the robustness of cross‐dataset conclusions, we indicate the core experimental and technical parameters of each dataset (Table 2). Before the sequencing step, these studies utilized various flow cytometry sorting techniques based on either endogenous fluorescent protein expression or specific epitopes for antibody‐based purification. They also included a range of different mouse strains, ages, and a range of cell numbers, yielding a diverse set of data for a comparative analysis. Although the tissue dissociation method was similar across studies, other technical differences, including variations in sequencing platform and data preprocessing methods, could in principle contribute to differences in the observed cellular composition and gene expression profiles. For instance, the Shin et al. (2015) dataset contains orders of magnitude fewer cells than the Harris et al. (2021) dataset, potentially affecting sensitivity in identifying shared markers (Tosoni et al. 2023). To mitigate this, we focused on markers consistently detected across datasets rather than those unique to a single study. Importantly, sequencing depth was not reported in most of the original publications we included. As sequencing depth is an important technical characteristic for sc/snRNAseq studies, we recommend it should be consistently documented. While certain dataset‐specific effects cannot be entirely ruled out, our approach highlights robust and recurrent signatures of NSC dynamics.

**TABLE 2:** Summary of single‐cell RNA sequencing (scRNA‐seq) datasets used in this study.

Study	Year	Animal strain	Age	Sex	Tissue processing and dissociation method	Cell sorting and enrichment strategy	Sequencing platform	Nb. of cells
Shin et al.	2015	Nestin CFPnuc	8–12 weeks	M	Microdissected DG, digested with papain	CFP+ single cell pickup using micromanipulator	Smart‐seq2	142 (CFPnuc+) + 26 (CFPnuc‐)
Artegiani et al.	2017	C57BL6, Nestin‐GFP	6 and 10 weeks, over 1 year	F+M	Microdissected DG, papain or trypsin neural‐tissue dissociation kit (Milteny Biotec)	GluR1‐/Cd24‐ double‐negative cells	SORT‐seq	1408
Batiuk et al.	2020	C57BL/6J	P56	F+M	Dissected hippocampus, digested with papain	ACSA‐2‐PE‐positive/anti‐O1‐eFluor660‐negative/7‐AAD negative	Smart‐seq2	2015
Bielefeld et al.	2024	Nestin‐GFP	8 weeks	M	Microdissected DG, Neural Tissue dissociation kit (Milteny Biotec)	GFP+ cell sorting with BD FACSAria III	10X	7791
Harris et al.	2021	Ki67‐TD, Nestin‐GFP	1, 2, 6–8 months	F+M	Microdissected DG, Neural Tissue dissociation kit (P) (Milteny Biotec)	Gated for tdTomato expression according to a control mouse that expressed Nestin‐GFP alone	10X	24,203 (2947 NSCs)
Hochgerner et al.	2018	CD1, hGFAP‐GFP	P0–P132	F+M	Microdissected DG, digested with papain	GFP+ cell sorting with BD FACSAria II	10X	Dataset A: 5454; Dataset B: 2303; Dataset C: 24185 (165 RGLs, 88 nIPCs)
Wu et al.	2025	C57BL/6JRj	3, 9–11, and 16–21 months	F+M	Microdissected DG, Neural Tissue dissociation kit (P) (Milteny Biotec)	No sorting	10X	34,732 (696 qNSC, aNSCPC, NB)

Study	Alignment/mapping	QC filtering	Normalization	Clustering
Shin et al.	Aligned to custom genome (which contained Nestin enhancer + eCFP) by RSEM (Li and Dewey 2011)	Outliers removal	RSEM	Distance matrix based on Pearson correlation between each pair of single cells
Artegiani et al.	Aligned to mouse transcriptome by BWA (Li and Durbin 2009)	Removal of cells with < 1500 (for clustering)/4000 (for DEG analysis) transcripts removed	Downsampling	RaceID2, Waterfall
Batiuk et al.	Aligned to GRCm38 (+ ERCC sequences)	FASTQC; < 54 transcripts removed	ln‐normalization (Seurat)	Seurat
Bielefeld et al.	Aligned to GRCm38/mm10 and refdata‐cellranger‐mm10‐3.0.0 by CellRanger v3.0.2	Removal of cells with degraded RNA or doublets	LogNormalize (Seurat)	Seurat
Harris et al.	Aligned to custom genome (mm10‐3.0.0 + eGFP, tdTomato, WPRE, bGHpolyA sequences) by CellRanger v3.0.2	Doublet removal (marker‐based); removal of cells < 500 genes, > 10% mitochondrial reads	SCTransform (Seurat)	Seurat
Hochgerner et al.	Aligned to reference genome by CellRanger v1.1 (Dataset A), v1.3 (Dataset C)	Doublet removal; > 600 (C:> 800) total genes, between 800 and 20,000 (C: 1000–30,000) molecules, and a ratio of molecules to genes > 1.2	Molecule counts per cell normalized to 10,000 (Dataset A)/5000 (Dataset C)	Markov clustering
Wu et al.	CellRanger + Seurat v4.3	Doublet removal by DoubletFinder; removal of cells < 800 genes, > 20% mitochondrial reads	NormalizeData, SCTransform (Seurat)	Seurat

**FIGURE 1:**
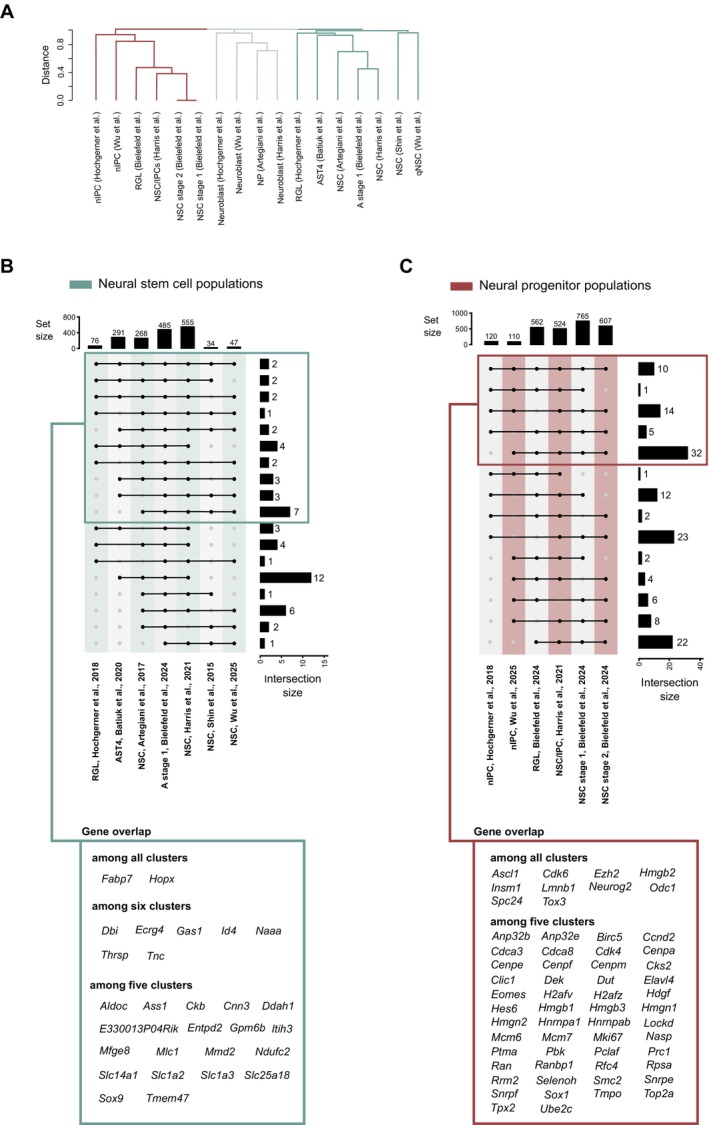
Comparison of gene expression profiles in adult hippocampal neural stem cell (NSC) and neural progenitor (NP) populations across single‐cell RNA seq (scRNA‐seq) studies. (A) Hierarchical clustering based on gene sets used to identify NSC, NP, or neuroblast cells across different scRNA‐seq studies. (B, C) Upset plot illustrating the overlap of expressed genes used to identify NSC (B) and NP (C) populations between scRNA‐seq studies. Analyses reveal significant overlap and distinct clustering patterns among the NSC and NP populations, highlighting the robustness of gene signatures across studies.

To group these populations in an unbiased way, we first applied hierarchical clustering using a similarity matrix based on their gene lists, which organizes populations based on the similarity of their gene expression profiles (Section 4). This method generated a dendrogram that visually represents the relationships between these populations (Figure 1A). Our first combined analysis of all the seven datasets revealed an effective separation into three main clusters, largely corresponding to the NSC, NP, or neuroblast annotations used in each study (Figure 1A). Interestingly, this analysis clustered together populations with different annotations, such as neuronal intermediate progenitor cells (nIPCs), RGLs, intermediate progenitor cells (IPCs), and NSC stage 1/2, demonstrating the divergent nomenclature of transcriptionally similar cell types (Figure 1A). On the other hand, the label neuroblast seems to be assigned more consistently across studies (Figure 1A). As an example of the practical application of our approach, the astrocytic cluster AST4 from Batiuk et al. (2020), which was hypothesized by the authors to be either a population of hippocampal NSCs or NPs, appears to be more closely linked to the NSC clusters of other studies, supporting the conclusion they may represent NSCs. Additionally, the NSC populations from Bielefeld et al. (2024) clustered closer to the NSC/IPCs cluster from Harris et al. (2021), suggesting that these cells may represent an intermediate or progenitor‐like state. Similarly, the astrocytic population A stage 1 in Bielefeld et al. (2024) clustered close to the NSC populations from Harris et al. (2021) and Artegiani et al. (2017) indicating they may represent NSCs. Therefore, our approach provides an example of how unbiased hierarchical clustering could be used to clarify relationships between cell populations identified across different studies and improve comparability between them, particularly given the current lack of a uniform annotation nomenclature.

To further investigate the similarities within similar clusters across studies, we conducted a comparison of the individual gene lists used to identify them in each study. Our analysis revealed a substantial overlap of genes shared among cell clusters identified in at least five studies for NSCs or among at least three studies for the NPs and neuroblasts (Figure 1B,C, Figure S1). Nevertheless, a total of 28 genes for NSCs in at least 5 and 62 genes for NPs in at least 3 studies were the same. Interestingly, only two genes were observed in common across the seven studies within NSCs (*Fabp7*, *Hopx*). Conversely, 10 genes (*Ascl1, Cdks6, Ezh2, Hmgb2, Insm1, Lmnb1, Neurog2, Odc1, Spc24, Tox3*) were detected in NPs from all studies (Figure 1B,C), suggesting these could be bona fide cell type‐specific markers.

Many of the genes that we found shared between multiple NSC populations have been previously associated with the maintenance of quiescence in NSC, such as *Id4* (Blomfield et al. 2019), *Hopx* (Shin et al. 2015; Berg et al. 2019), and *Aldoc* (Shin et al. 2015) or are linked to the negative regulation of cell proliferation, such as *Sox9* (Scott et al. 2010) and *Ecrg4* (also known as *1500015ORik*) (Tang et al. 2019), suggesting that annotated “NSC” populations in these studies may represent qNSCs. Within the genes shared by NSC populations, we also found genes associated with L‐glutamate transport, such as *Slc1a3* (also known as *Glast1* or *Eaat1*), *Slc1a2* (also known as *Eaat2*), and *Slc25a18* (Figure 1B). L‐glutamate transporters have been implicated in various aspects of NSC behavior, particularly in regulating self‐renewal and influencing the proliferation and differentiation of immature neural cells (Schlett 2006; Rieskamp et al. 2023). Within the NP populations, we observed an overlap of genes associated with cellular processes, such as cell cycle regulation (e.g., *Ccnd2, Cdk4, Mcm6, Mcm7*), centromere function and chromosome segregation (e.g., *Cenpa, Cenpe, Cenpf, Cenpm*), and the regulation of neurogenesis (e.g., *Ascl1, Hes6, Ezh2, Hmgb2*), perhaps reflecting NSC activation (Figure 1C). Particularly noteworthy is the presence of *Ascl1* and *Vim*, which are shared among four different studies included in our comparison, as *Ascl1* and *Vim* have been widely used as NSC markers (Bottes et al. 2021; Encinas et al. 2011). These observations indicate that some of the cell populations annotated as “NPs” across studies may rather represent aNSCs, thereby providing another example of how scRNA‐seq data reanalysis across studies could help to provide a more accurate classification of cell populations.

Several genes (*Hopx, Id4, Aldoc, Entpd2, Gpm6b, Itih3, Mfge8, Mlc1, Mmd2, Slc14a1, Slc1a2, Slc1a3, Slc25a18, Sox9, Tmem47*) that were shared between at least five NSC clusters (Figure 1B) were also enriched in juvenile/adult V‐SVZ and SGZ NSCs, relative to embryonic NSCs and juvenile/adult V‐SVZ transit‐amplifying cells and SGZ intermediate progenitors (Borrett et al. 2022). However, many of these genes are also expressed in astrocytes, as demonstrated by similar gene abundance levels in SGZ NSCs versus SGZ astrocytes in the same study (Borrett et al. 2022), making them unsuitable for the purpose of a clear identification of NSCs and, in particular, their discrimination from astrocytes.

**FIGURE 2:**
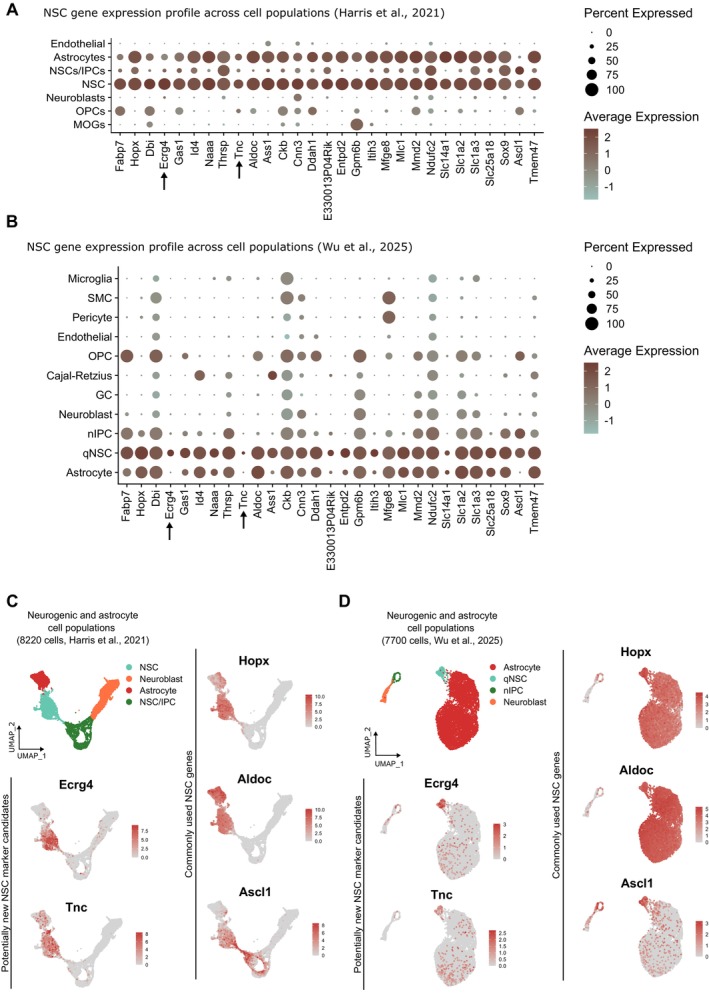
Cell‐type specific expression of Ecrg4 and Tnc across single‐cell RNA sequencing (scRNA‐seq) datasets. (A, B) Dot plot showing gene expression of shared NSC genes across cell populations in the Harris et al. (2021) dataset (A) and in the Wu et al. (2025) dataset (B). (C, D) UMAP plots depicting the expression of *Ecrg4, Tnc, Hopx, Aldoc*, and *Ascl1* across NSCs, Neuroblasts, Astrocytes, and NSC/IPC clusters in the Harris et al. (2021) dataset (C) and across Astrocytes, qNSC, nIPC, and Neuroblasts in the Wu et al. (2025) dataset (D).

Although our first analysis readily identified genes highly conserved across different studies (*Fabp7*, *Hopx, Dbi, Ecrg4, Gas1, Id4, Naaa, Thrsp*, and *Tnc* in NSC populations), this does not necessarily mean that they are exclusively expressed in specific populations. To further investigate the specific expression profiles of these shared genes and identify potential new markers, we assessed their distribution across cell types in the datasets of Harris et al. (2021) and Wu et al. (2025). By using dot plots visualization (Section 4), we showed how gene expression varies across different clusters. In these plots, the size of the dot encodes the percentage of cells within a class, while the color encodes the average expression level across all cells within a class (Section 4). Our comparison reveals that *Ecrg4* and *Tnc* exhibited the most restricted expression profiles in NSCs compared to other cell populations (Figure 2A,B). To further illustrate this, we visualized their expression on UMAPs of the NSC, NPs, neuroblast, and astrocyte clusters from both studies (Figure 2C,D and Section 4). Compared to commonly used markers such as *Hopx, Aldoc*, and *Ascl1*, *Ecrg4* and *Tnc* appear more specific to NSCs, with lower expression in astrocytes, NPs, and neuroblasts. However, *Tnc* expression is relatively low and restricted to a smaller proportion of NSCs. Among these candidates, *Ecrg4* might thus be a promising marker for DG NSCs due to its more specific expression pattern.

In addition to their specific expression patterns, these genes play important functional roles in neurogenesis. *Ecrg4* has been shown to be expressed in NSC niches, and its deficiency was able to prolong NSC proliferation activity and improve cognitive function (Nakatani et al. 2019). Additionally, *Ecrg4* acts as a senescence inducer in neural precursor cells, further supporting a functional contribution in NSC homeostasis and aging (Kujuro et al. 2010). Similarly, *Tnc* encodes for Tenascin‐C, an extracellular matrix protein that has important roles in cell cycle regulation and neural stem/progenitor cell motility (Schaberg et al. 2022). Tenascin‐C is highly localized in neurogenic niches such as the SVZ and hippocampus and has been implicated in neurogenesis (Faissner et al. 2017; Garwood et al. 2004; Tucić et al. 2021).

### 2.3 Functional Insights From Gene Expression Patterns and Pseudotime Inference Analysis of NSC and NP Populations

Besides gaining insights into the transcriptomic landscape of NSCs and NPs, the continuous improvement of bioinformatic pipelines allows for the dynamic sorting of cell populations along an in silico‐computed temporal trajectory or pseudo‐temporal axis. Pseudotime is a computational technique used to order cells based on their gene expression profiles, creating a “simulated” timeline that reflects the likely progression of cellular states through developmental processes, such as differentiation (Cannoodt et al. 2016). Unlike chronological time, pseudotime represents a theoretical trajectory that captures the dynamic transition between different cellular “states” of distinct transcriptional signatures along a differentiation “continuum.” This method allows to move beyond static snapshots of transcriptional profiles in NSCs, providing a more nuanced view of their molecular diversity. It is important to note that pseudotime is not a vector and does not inherently indicate directionality, unlike RNA velocity, which can estimate the future transcriptional state of NSC based on spliced and unspliced mRNA ratios (Bielefeld et al. 2024).

**FIGURE 3:**
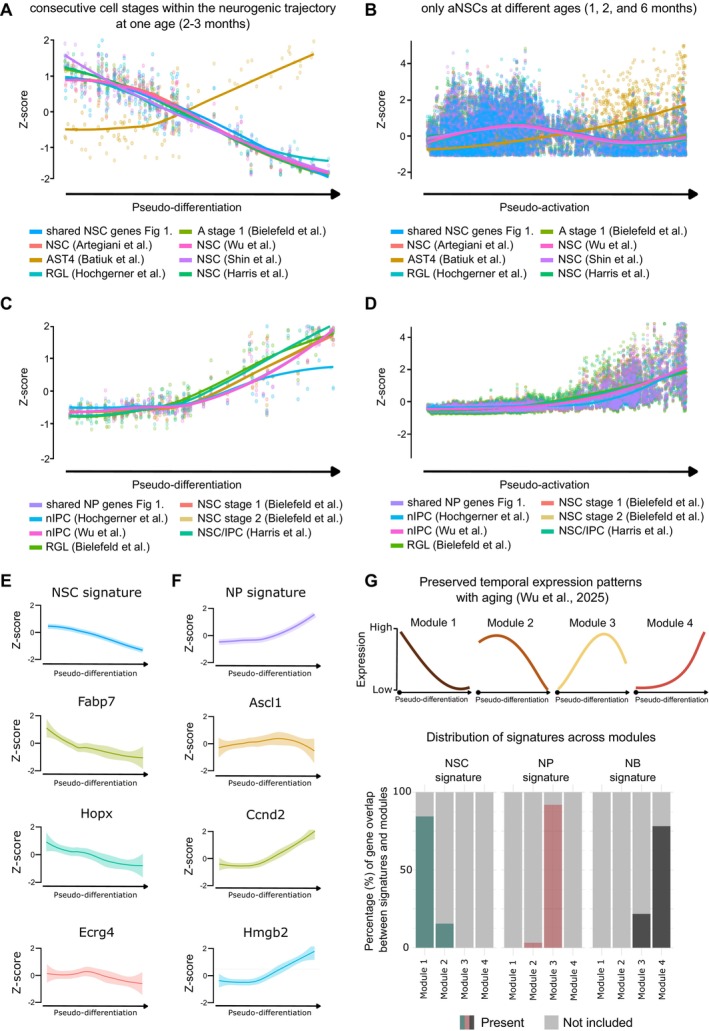
Pseudotime gene expression patterns across adult neural stem cell (NSC) and neural progenitor (NP) populations across multiple studies. (A, C) Pseudotime trajectories derived from the Shin et al. (2015) dataset using the Waterfall algorithm, depicting all cell clusters from the compared studies. (B, D) Pseudotime trajectories from the Harris et al. (2021) dataset using the Slingshot tool, showing all cell clusters from the compared studies. (E, F) Gene expression dynamics in the Shin et al. (2015) dataset, showing the expression of individual genes as well as the NSC and NP signatures across pseudotime. (G) Overlap of the amount of genes in the NSC and NP gene signatures found within the age‐conserved gene modules from Wu et al. (2025), which span 3‐, 9–11‐, and 16–21‐month‐old mice. Modules 1 and 2 correspond to early stage NSC identity, while Modules 3 and 4 are linked to later differentiation stages. The variability in pseudotime trajectory of the various gene sets further emphasizes the need for a nuanced understanding of the biological processes underlying neural differentiation and the relevance of core gene sets in capturing these transitions.

By mapping cells along this pseudotemporal axis, we can observe how NSCs transition from quiescence to full activation. Using as reference two publicly available datasets with pseudotime information, focusing on NSC in the adult hippocampus (Harris et al. 2021; Shin et al. 2015), we examined whether distinct patterns in activation states could be observed across the NSC and NP populations described in the different scRNA‐seq datasets we compared. The Shin et al. (2015) dataset employed the Waterfall algorithm to calculate pseudotime, capturing the progression of subsequent cell stages within the neurogenic trajectory at a single age (indicated as pseudo‐differentiation; Figure 3A,C). In contrast, the Harris et al. (2021) dataset used the trajectory inference tool Slingshot to model NSC states across different ages (indicated as pseudo‐activation; Figure 3B,D). For each dataset, we plotted the *z*‐score normalized average expression of genes used to identify NSCs and NPs along the pseudotime trajectory (Section 4). This allowed us to visualize expression patterns and compare profiles between studies. The core gene sets identified as shared among at least five NSC clusters or NP clusters in Figure 1B,C were compared against the original gene sets used for identifying the individual populations in each study.

Interestingly, the shared core gene sets exhibited expression patterns that were strikingly similar to those of the individual gene sets (Figure 3A–D). This consistent trend across different datasets reinforces the validity of these overlapping gene sets as reliable markers for NSC and NP profiling. Despite the two different informatic tools used for pseudotime analysis, NSC and NP populations showed similar trends across studies yet maintained distinct profiles from each other. More specifically, NSCs initially showed a higher gene expression that declines along the temporal trajectory. Of note, NP populations exhibited a reverse pattern: initially lower gene expression that increased over pseudotime (Figure 3B,D).

Hence, pseudotime depiction correctly represented the expected expression dynamics of those populations, with NSCs exhibiting higher expression profiles of genes associated with a quiescent state, and NPs exhibiting higher profiles of genes associated with activation. Interestingly, subtle differences between studies are evident, for example, the nIPC cluster from Hochgerner et al. (2018) shows an almost stable expression in the first half of the pseudotime trajectory that increases afterward (Figure 3C,D). In contrast, other NP signatures start increasing earlier along the pseudotime trajectory. Notably, the Hochgerner et al. (2018) dataset includes cells from a different wild‐type mouse strain (CD1) compared to the other studies (C57BL6), which may contribute to the observed differences. While mouse strain could be a potential confounder, other biological or technical variables, like sex, environmental conditions, and differences in experimental procedures, may also play a role. Therefore, it is important to consider the influence of strain alongside other possible factors when interpreting findings across different studies. Interestingly, the astrocytic cluster AST4 from Batiuk et al. (2020), which appeared to be closely linked to the NSC clusters from other studies in our hierarchical clustering analysis (Figure 1A), showed different pseudotime differentiation and activation profiles than the majority of other populations, indicating intrinsic differences that separate them from populations that clustered close together, such as NSC from Artegiani et al. (2017), A stage 1 cells from Bielefeld et al. (2024), NSC from Harris et al. (2021), or RGL from Hochgerner et al. (2018) (Figure 1A), supporting our conclusion that pseudotime analysis can be instrumental in differentiating closely related cell populations.

To further validate the relevance of our proposed core gene signatures, we examined the expression dynamics of selected genes along the pseudotime trajectory, including genes associated with quiescence (e.g., *Hopx*) and activation (e.g., *Ascl1*) (Figure 3E,F). This analysis provides additional support for the robustness of our core gene sets, as their expression profiles align well with expected biological transitions. Although individual gene expression can be variable, these profiles reinforce the validity of our identified NSC and NP gene signatures.

Furthermore, we investigated a potential age‐related bias in gene overlap across datasets. For this, we utilized the age‐conserved gene modules defined by Wu et al. (2025), spanning 3, 9–11, and 16–21 months old mice, which represent stage‐specific transcriptional programs, with Modules 1 and 2 corresponding to early stage NSC identity and quiescence, and Modules 3 and 4 linked to later differentiation stages (Figure 3G). Strikingly, we found that all NSC genes were mapped to Modules 1 and 2, which are associated with early stage NSC identity and quiescence, reinforcing their conserved role across aging. Similarly, the NP signature predominantly aligned with Module 3, with only three genes not captured in any module (Figure 3G). Finally, all genes of the neuroblast signature were allocated to Modules 3 and 4. This strong correspondence between our findings and the age‐preserved transcriptomic modules described in Wu et al. (2025) suggests that the identified shared genes are not merely a result of dataset intersection bias but rather represent a core, conserved transcriptional program underlying NSC and NP states across the adult lifespan (Figure 3G).

There are some limitations to the pseudotime‐based analyses we present here. The linear trajectory assumed in the pseudotime analysis (i.e., from quiescence to activation) may not fully capture the complex behavior of NSCs/NPs, as late differentiation and other downstream cellular processes are not included. Additionally, while using a *z*‐score for normalization aids their comparison, integrating datasets and comparing them collectively would provide a higher resolution and a more comprehensive understanding of the gene expression dynamics. Drawbacks of this approach include the requirement of higher computational power and the possibility that differences in study design across datasets and in their analysis might not reflect biological chances but rather technical ones. Nevertheless, pseudotime analysis is useful to reveal the molecular similarity within NSC or NP populations across studies, as well as the diversity and temporal progression of NSCs versus NPs. Finally, it highlights the importance of considering methodological and biological variability in such studies.

In summary, our comparative reanalysis of seven recent scRNA‐seq studies provides valuable insights into the molecular signatures of NSCs and NPs in the hippocampus. Importantly, our cross‐analysis identified commonalities across studies, facilitating comparisons and indicating which populations may be more similar to each other and which gene sets may be used as future population markers. Across variations in mouse models and ages used in the studies, our analysis revealed overlapping genes associated with key cellular processes such as cell cycle regulation, translation, and neurogenesis regulation. Some genes showed potential as new specific markers for NSCs, namely *Ecrg4* and *Tnc*. Remarkably, some of the genes we identify in our cross‐study data reanalysis have been used as markers to identify NSCs and NPCs (e.g., *Fabp7* [also known as *BLBP*], Hopx, Table 1) or their promoter sequences used to, for example, drive fluorescent protein expression in transgenic reporter mouse lines commonly used to label NSCs (e.g., *Slc1a3/Glast1*), but their expression is distributed across several cell types (Figure 2A,B). However, additional studies should be conducted to investigate the validity and specificity of the new gene sets identified in our analysis for their use as NSC markers or in reporter mouse lines. Hopefully, those studies will then help to refine the classification of NSCs and better understand their heterogeneity and how they differ from closely related and sometimes overlapping cell populations, such as NPs.

Using pseudotime analysis, we have delineated distinct activation patterns within NSC and NP populations across various studies. This offers valuable insights into the molecular diversity and temporal dynamics of NSCs and NPs, laying the groundwork for further refinement in their classification and understanding. Although immunohistochemical methods have been instrumental in characterizing NSC heterogeneity, recent advancements in scRNA‐seq have opened new avenues for a deeper exploration of this cell diversity with respect to possible different functions within the complex microenvironment of the neurogenic niche.

However, scRNA‐seq introduces its own set of challenges. Cell purification methods based on the expression of fluorescent proteins driven by specific promoters and the antibodies used for fluorescence‐activated cell sorting can introduce biases, potentially leading to misinterpretation of results. Moreover, the choice between snRNA‐seq and scRNA‐seq requires careful consideration. For example, snRNA‐seq may be preferred in scenarios where obtaining intact single cells is challenging, but it may yield different results compared to traditional scRNA‐seq, such as variations in cellular context, sampling bias toward certain cell types, RNA degradation effects, and differential expression detection capabilities (Habib et al. 2017). Furthermore, the diversity of pipelines and computational tools for scRNA‐seq analysis introduces challenges in standardization among groups and may impact the reproducibility and comparability of results.

### 2.4 A Transcriptomic‐Based Framework to Study NSCs and Aging

The brain's ability to generate new neurons throughout adulthood, has been observed to markedly decline with age, coincident with decreased hippocampus‐dependent cognition and the brain's capacity for memory, cognition, and mood regulation, as shown in studies conducted in mice and rats (Harris et al. 2021; Martín‐Suárez et al. 2019; Kuhn et al. 1996; Kempermann et al. 1998; Heine et al. 2004; Gil‐Mohapel et al. 2013). The progressive loss of, in particular, NSCs is a major factor for the age‐related decline of neurogenesis (Encinas et al. 2011). This decline is further aggravated by age‐dependent changes intrinsic to NSCs and the micro‐ and macro‐environment surrounding them (Kempermann et al. 1998; Okamoto et al. 2011; Marlatt et al. 2012; DeCarolis et al. 2015; Cole et al. 2022).

**FIGURE 4:**
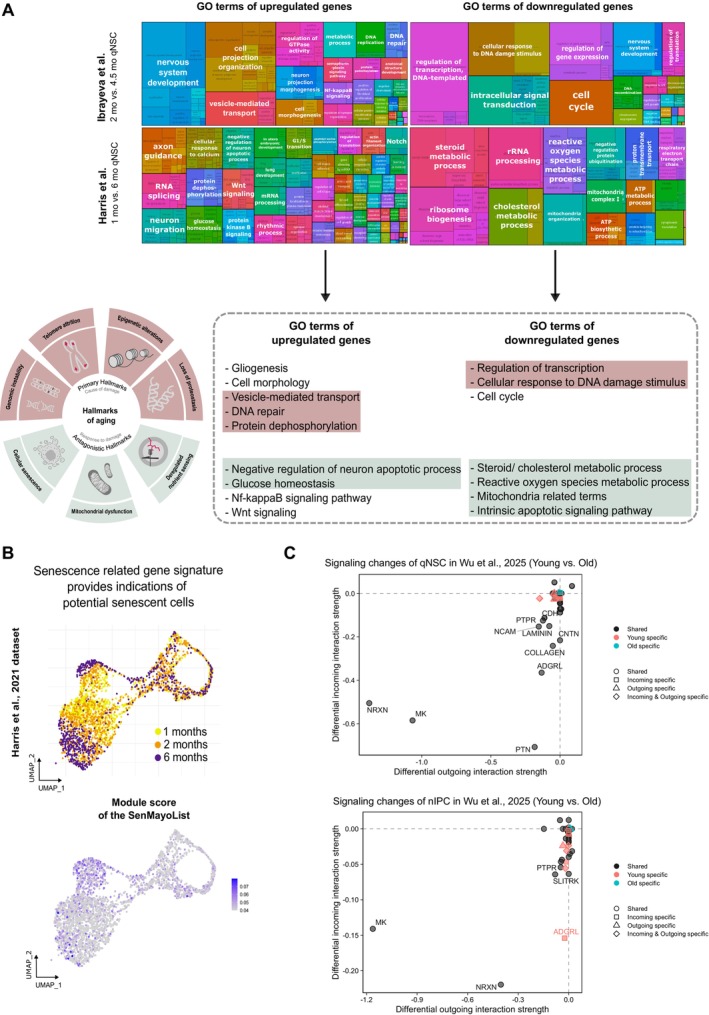
Aging characteristics in older quiescent neural stem cells. (A) Gene Ontology (GO) term summary based on differentially expressed genes of aged adult neural stem cell (NSC) comparisons from Ibrayeva et al. (2021) and Harris et al. (2021). Hallmarks of aging adapted from Vujin and Dick (2020). (B) ModuleScore of the SenMayo gene list (125 senescence‐related genes) on the NSC and neural progenitor (NP) populations of Harris et al. (2021), comprising 1, 2, and 6 months old cells. (C) Changes in signaling pathways of qNSCs and nIPCs from young (3 months) and old (16–21 months) cells of the Wu et al. (2025) dataset using CellChat. Results illustrate the aging characteristics of NSCs, revealing significant age‐related changes in cellular pathways. The SenMayo ModuleScore highlights an increase in senescence‐associated gene expression with age, underscoring a possible role of senescent NSCs in the aging process.

The insights gained from scRNA‐seq dataset comparisons can offer a valuable framework to explore age‐related changes in NSCs. Indeed, we found two studies that investigated the transcriptional landscape in NSCs during aging (Ibrayeva et al. 2021; Harris et al. 2021). By using gene ontology term analysis, both studies show changes in neurogenesis, gliogenesis, and cell cycle (summarized in Figure 4A) supporting the notion that aged NSCs behave differently regarding their functional dynamics (including reactivation and differentiation into other cell types). Biological functions driving cellular aging were further identified as being overrepresented in NSCs as early as 4.5 months old (Ibrayeva et al. 2021). These pathways included epigenetic dysregulation characterized by histone demethylation, downregulation of transcription, and inflammation linked to NIK/NF‐kappaB signaling. Additionally, changes in metabolism and proteostasis, alongside cellular stress resulting from loss of DNA recombination and DNA repair mechanisms, may contribute to a functional decline in which NSCs exhibit reduced proliferation, differentiation, or responsiveness due to both intrinsic and extrinsic factors—processes consistent with established hallmarks of aging (López‐Otín et al. 2013). Similar changes were described by Harris et al. (2021) and long‐lasting changes in these biological functions during aging could indeed lead to cellular senescence of NSCs, an irreversible cell proliferation state distinct from functional decline.

Cellular senescence is a multifactorial state in which cells permanently cease to proliferate and undergo distinct phenotypic changes, including alterations in gene expression, morphology, and function. ANSCs may undergo replicative senescence due to reaching their proliferative limit from telomere shortening, as evidenced by findings in telomerase‐deficient mice, where telomere shortening in NSCs of the SVZ negatively impacts neuronal differentiation and contributes to a decline in neurogenesis (Ferrón et al. 2009). However, a less well‐investigated hypothesis is that age‐dependent cell‐intrinsic alterations take place in (a proportion of) qNSCs, allowing them to switch from a quiescent to senescent state.

NSCs can express a senescent phenotype including important biomarkers such as an upregulation of cell cycle inhibitors p16, p21, and p53, increased activity of SA‐β‐gal, and loss of lamin‐B1. Also, DNA damage indicated by γH2AX and the activation of mTOR signaling pathways have been observed to markedly increase with age (Molofsky et al. 2006; Bin Imtiaz et al. 2021; Fatt et al. 2022). Just like the characterization of NSCs, defining senescence in vitro and in vivo lacks universal or senescence‐specific markers (Hernandez‐Segura et al. 2018).

To further investigate senescence signatures within NSCs, we used the scRNA‐seq dataset from Harris et al. (2021) and the SenMayo gene list (Saul et al. 2022), comprising 125 genes associated with cellular senescence. This gene set consists predominantly of senescence‐associated secretory phenotype (SASP) factors (*n* = 83), but also transmembrane (*n* = 20) and intracellular (*n* = 22) proteins. We calculated a module score for the NSC and NP cluster within the dataset, at a stringent cutoff of 0.4. The analysis revealed a subtle yet higher module score in the UMAP around the 6‐month‐old cells compared to 1‐month‐old cells (Figure 4B), suggesting NSCs present in older mice express a higher proportion of senescence‐associated genes. This analysis exemplifies another way in which such scRNA‐seq tools could offer more insight and a systematic approach to address the difficulties associated with the study of cellular senescence.

As mentioned before, different NSC morphophenotypes exist, of which their relative proportions increase with age in the DG. Type ꞵ‐ and Ω‐cells are non‐proliferative and mostly astrogliogenic, suggesting qNSCs may transform into astrocytes (Gebara et al. 2016; Martín‐Suárez et al. 2019). Several studies have reported that NSCs become more deeply quiescent during aging and thereby become more resistant to activation and induced proliferation (Ibrayeva et al. 2021; Harris et al. 2021; Martín‐Suárez et al. 2019). Furthermore, qNSCs have been reported to display various age‐related intrinsic alterations, such as impaired proteostasis, accompanied by dysfunctional lysosomes and an accumulation of protein aggregates, as well as a decrease in autophagy and changes in mitochondrial metabolism, which are common molecular mechanisms resulting in cellular senescence in other stem cells (García‐Prat et al. 2016; Revuelta and Matheu 2017; Leeman et al. 2018; Wang et al. 2023).

Another mechanism through which senescent NSCs may impact the hippocampus is by promoting the formation of a pro‐inflammatory microenvironment via their SASP. This SASP is thought to be the primary mediator of paracrine effects of senescent NSCs. SASP components reduce proliferation and promote inflammatory responses in non‐senescent NSCs within the stem cell niche, further reducing the capacity for regeneration of the adult brain (Kalamakis et al. 2019). Indeed, induction of NSC senescence resulted in an increase in the expression of inflammatory mediators and reactive oxygen species‐mediated pathways (Gasperini et al. 2023). Moreover, SASP components (e.g., CCL3, granulocyte–macrophage colony‐stimulating factor, IL‐2, and IL‐27) that recruit immune cells for immunosurveillance may contribute to neuroinflammation and subsequent decline in neurogenesis, as evidenced by the accumulation of natural killer (NK) cells in the murine and human DG neurogenic niche during aging, where they are in close proximity to DCX+ DG cells (Jin et al. 2021). More recently, age‐induced widespread transcriptomic changes in the DG were strongly associated with T‐cell‐mediated inflammatory responses. Notably, they observed increased T‐cell infiltration within the DG, suggesting that immune infiltration may play a crucial role in modulating the neurogenic niche (Wu et al. 2025).

Intriguingly, senescent NSCs overexpress IL‐33 (Gasperini et al. 2023), which mediates neuroinflammation‐induced hippocampus‐dependent cognitive impairment (Reverchon et al. 2020). IL‐33 further improves NSC proliferation after a hypoxic insult (Tian et al. 2023). In line with this, IL‐15, another interleukin component of the senescent NSCs SASP (Gasperini et al. 2023), is associated with increased proliferation in the DG under basal conditions in IL‐2 knockout (IL‐2 KO) mice (Beck Jr et al. 2005), suggesting that the senescent NSC SASP may promote NSC proliferation. However, this increase in proliferation was specifically observed in IL‐2 KO mice and only in males, indicating context‐dependency and a potential sex‐specific effect. Interestingly, immature neurons in the DG express IL‐27, which drives NK cell infiltration, activation, and cytotoxicity and in turn, can induce inflammation and neurodegeneration (Jin et al. 2021). This could provide a possible explanation as to how senescent NSCs may promote neuroinflammation and neurodegeneration via paracrine effects mediated by their SASP.

To further investigate how aging might affect cellular communication in the neurogenic niche, we performed CellChat analysis (Section 4) on the young (3 months) and old (16–21 months) datasets from Wu et al. (2025). Our results reveal a global reduction in both the number and strength of interactions between cell types in the older environment (Figure S2A). This decline is observed across multiple cell types, consistent with an overall weakening of the neurogenic niche's capacity to support NSC function. Notably, the only predicted interactions that increased in strength with age were between endothelial cells and neuroblasts, as well as pericytes and neuroblasts (Figure S2B), suggesting potential compensatory mechanisms in vascular‐associated signaling or a higher dependency from vascular factors in aged neuroblasts. A closer examination of signaling pathways within neurogenic populations revealed specific alterations, including changes in neurexin (NRXN), midkine (MK), pleiotrophin, and protein tyrosine phosphatase receptor‐mediated pathways (Figure 4C, Figure S2C). NRXN signaling, which plays a critical role in synaptic organization, was reduced, potentially contributing to the weakened cell–cell interactions observed in the aging neurogenic niche (Südhof 2017). MK and PTN pathways, both involved in neuroprotection and NSC maintenance, also showed significant alterations, which may reflect a disrupted regenerative environment (Winkler and Yao 2014). Furthermore, we observed changes in neural cell adhesion molecule (NCAM), laminin, and collagen signaling specifically within qNSCs. NCAM is crucial for cell–cell adhesion and neurogenic lineage progression, while laminin and collagen contribute to extracellular matrix integrity and niche stability (Shin et al. 2002; Chen et al. 2003; Frantz et al. 2010). Alterations in these pathways suggest an age‐related decline in structural and adhesion‐mediated support for qNSC maintenance, potentially contributing to their impaired activation. Together, these findings provide a more comprehensive view of how aging may affect cell–cell communication in the neurogenic niche, reinforcing the idea that both intrinsic and extrinsic factors contribute to NSC dysfunction.

**FIGURE 5:**
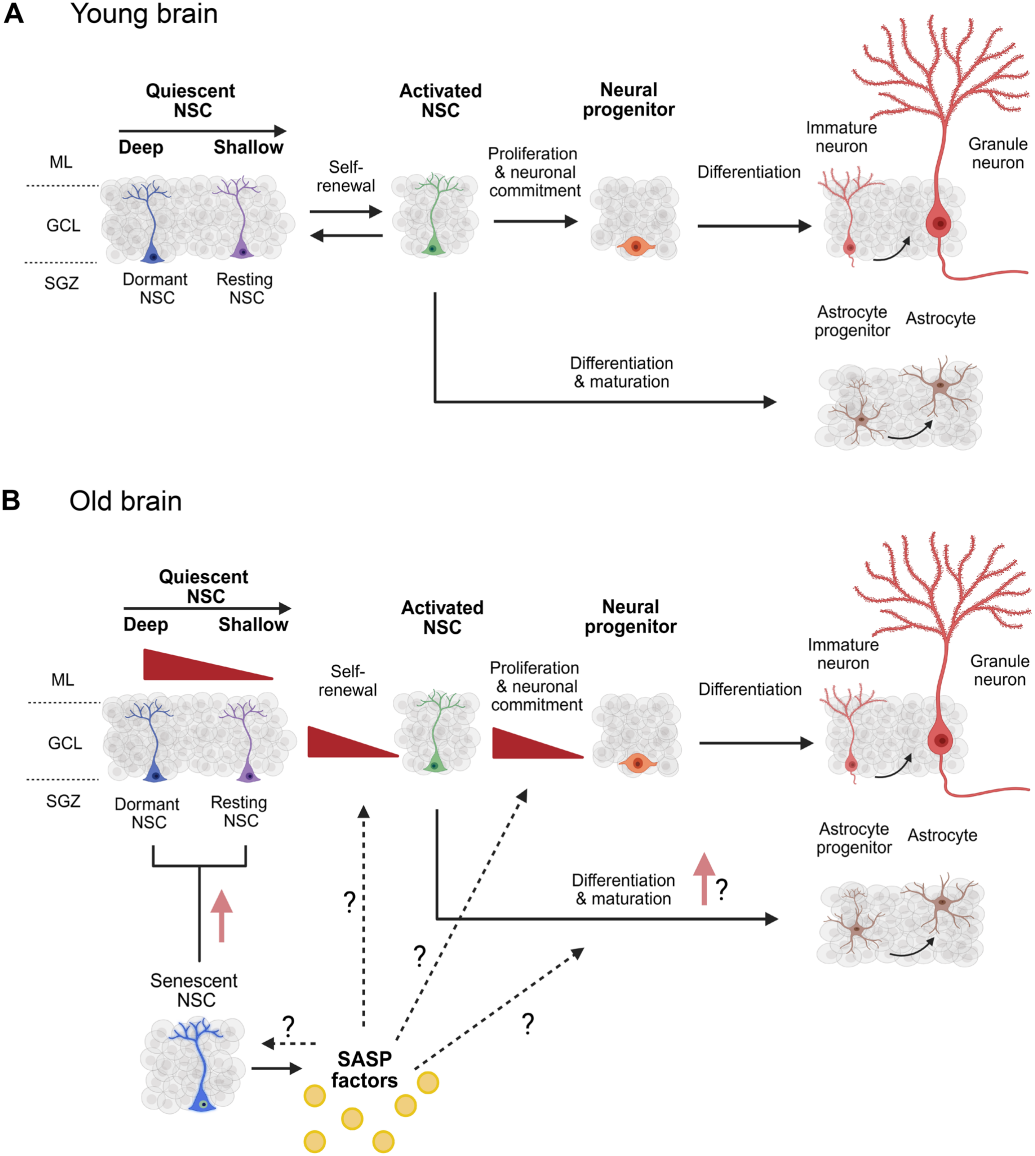
Schematic representation of neural stem cell dynamics in the young and aged brain. (A) Diagram illustrating the progression of adult neural stem cells (NSCs) in young brains from different levels of quiescence to activation, followed by differentiation into neural progenitor cells (NPs), and ultimately into neurons or astrocytes. (B) Similar schematic representing the same processes in the aged brain, with changes highlighted by red triangles. Senescent NSCs are introduced, and the potential impact of the senescence‐associated secretory phenotype (SASP) on these processes is indicated by dotted lines.

In summary, our analysis indicated that in the young brain, qNSCs can undergo proliferation in response to activation signals (Figure 5A). In the aged brain, however, not just the accumulation of non‐proliferative, senescent NSCs themselves, but also the local environment in the adult hippocampal stem cell niche may inhibit the regenerative capacity of the adult and aging brain (Figure 5B). By leveraging scRNA‐seq datasets to uncover the molecular pathways behind these intrinsic and environmental changes, innovative therapeutic strategies could be developed to help preserve neurogenesis with increasing age.

## 3 Conclusions and Future Directions

While the multiple dynamic fates and heterogeneous nature of NSCs present still challenges for classification, our cross‐analysis of scRNA‐seq highlights new opportunities for more precise identification of NSC subtypes. Advances in the field with novel techniques like sc/snRNA‐seq have provided higher resolution and coverage, revealing more and more the significant heterogeneity within NSC populations, which necessitates more refined classification strategies. By using gene expression profiles shared across datasets, we propose a novel set of markers that can be used to allow distinguishing “NSCs” from “NPs” and understand their intrinsic differences. This approach could resolve the current inconsistencies in cell‐type annotation, improving comparisons between studies and establishing a more uniform nomenclature.

However, several crucial questions remain unanswered: are NSCs “mother” cells, or do they contribute additional, non‐canonical, perhaps regulatory, functions to their local niche particularly as they age and become senescent? Do these roles persist in their progeny, and if so, do distinct lineages carry specific gene signatures that program their functionality? Do deeply qNSCs transform into senescent NSCs and promote a pro‐inflammatory environment that affects the function of the aged hippocampus? Addressing these questions could fundamentally alter our understanding of whether these cell populations represent unique functional cell types, or merely transitional states on the path toward more mature cell types. Furthermore, aging dramatically influences NSC behavior, adding another layer of complexity to their classification.

Future studies should consider integrating datasets for a unified analysis, leveraging advanced scRNA‐seq data integration and batch correction methods beyond traditional approaches. While the manual annotation of cell clusters based on historical markers carries a risk of circular logic, it remains a widely used approach, particularly when markers have been functionally validated beforehand. For example, in the mouse brain, key markers such as Sox2 and Hes5 for NSC, and Dcx and NeuroD1 for neuroblast differentiation, have been experimentally shown to play functional roles in these populations. However, an alternative approach would be to reanalyze and recluster the data in an entirely unsupervised manner to establish marker genes de novo. Techniques such as scANVI already offer powerful tools for harmonizing datasets while preserving meaningful biological variability (Xu et al. 2021; Luecken et al. 2022). A fully unsupervised re‐clustering followed by differential expression analysis could provide new insights into NSC pool heterogeneity, minimizing biases introduced by historical classifications and enhancing comparability across studies. Additionally, incorporating more advanced multi‐branch trajectory inference tools could help uncover alternative lineage pathways within the neurogenic niche. While our current analysis focused on the principal neurogenic trajectory, future work could explore whether certain subpopulations follow distinct developmental paths. Methods such as CellRank could offer deeper insights into fate decisions, capturing potential branching events that may not be apparent in traditional pseudotime models (Lange et al. 2022; Weiler et al. 2024).

Insights gained from (combined) sc/snRNA‐seq studies can help better delineate cell subtypes, such as senescent NSCs and potentially uncover novel targets for age‐related neurodegenerative conditions. Ultimately, a clearer understanding of NSC heterogeneity could help develop therapeutic strategies aimed at modulating neurogenesis in the aging brain.

## 4 Methods

### 4.1 Data Collection and Processing

Gene lists corresponding to adult NSCs, NPs, and neuroblasts were obtained from publicly available scRNA‐seq datasets (Harris et al. 2021; Artegiani et al. 2017; Bielefeld et al. 2024; Shin et al. 2015; Batiuk et al. 2020; Wu et al. 2025; Hochgerner et al. 2018). The selection criteria for genes included in the analysis were a fold change (FC) threshold of > 1.5 and an adjusted *p*‐value (*p*.adj) < 0.05. For one dataset, the FC threshold was 0.25 ln as per the original criteria. These lists were then compiled and formatted for downstream comparative analysis.

### 4.2 Hierarchical Clustering Analysis

Hierarchical clustering was performed based on gene set similarity across studies. A similarity matrix was generated by computing the number of common genes between each pair of datasets. The similarity matrix was converted into a distance matrix, which was used to perform hierarchical clustering using the hclust function from the stats package (version 4.4.1) in RStudio 2024.09.0, R 4.4.1.

### 4.3 UpSet Plot Visualization

To visualize gene set intersections across multiple datasets, UpSet plots were generated using the UpSetR package (Conway et al. 2017). UpSet plots were generated separately for NSCs, NPs, and neuroblasts, with each matrix summarizing the frequency of shared genes across studies. The UpSet function was employed to visualize gene intersections, and results were exported for further interpretation.

### 4.4 Gene Expression Profiles and UMAP Visualization

To analyze scRNA‐seq data and visualize gene expression patterns, we utilized Seurat (version 5.2.1). The Seurat objects from Wu et al. (2025) and Harris et al. (2021) were retrieved from the original study. Dot plots were generated using the DotPlot function, and UMAP projections were generated using the FeaturePlot function to visualize gene expression profiles across cell populations.

### 4.5 Pseudotime Analysis

Pseudotime analysis was performed using the pseudotime data provided by Shin et al. (2015) and Harris et al. (2021) from their respective datasets (Harris et al. 2021; Shin et al. 2015). From these datasets, we plotted the gene signatures and individual genes along the corresponding pseudotime trajectories. For the gene lists of each study, we used the *z*‐score normalized average gene expression of the identified genes. Additionally, genes from the age‐conserved modules defined by Wu et al. (2025) were extracted from the original paper and compared with our identified signatures to assess overlap (Wu et al. 2025).

### 4.6 GO Term Analysis

GO term lists were obtained from the respective studies (Ibrayeva et al. 2021; Harris et al. 2021). The extracted GO terms of young and old NSCs were then summarized using REVIGO (Supek et al. 2011), which reduces redundancy and provides a concise representation of enriched biological processes.

### 4.7 Senescence Module Score

To assess the enrichment of senescence‐associated gene signatures in a dataset, we calculated a module score using UCell (version 2.10.1) (Andreatta and Carmona 2021). Specifically, we utilized the function AddModuleScore_UCell() to quantify the relative expression of a predefined senescence‐related gene set. As the gene set, we employed the senMayo list, which comprises 83 SASP factors, 20 transmembrane proteins, and 22 intracellular proteins (Saul et al. 2022). The module score was computed for the NSC and NP clusters identified within the Harris et al. (2021) dataset, applying a stringent cutoff of 0.4 to define high‐scoring cells.

### 4.8 Cell–Cell Communication Analysis

Cell–cell communication analysis was performed using the CellChat R package (version 2.1.2) on the dataset of Wu et al. (2025), which includes scRNA‐seq data of young (3 months) and old (16–21 months) animals. Initially, the Seurat object was loaded, and the CellChat objects were created for each age group by subsetting. The CellChatDB.mouse was used to assign a pathway interaction database. The communication between cells was then visualized using the netVisual_aggregate function, which enables the depiction of cell–cell interactions in a circular network format.

## Author Contributions

O.P. collected and analyzed the data, prepared figures, and wrote the manuscript. E.K. wrote the manuscript and prepared figures in collaboration with O.P. P.J.L. contributed to project supervision and corrected the manuscript. E.S. supervised data analysis, contributed to project supervision, and corrected the manuscript. C.P.F. supervised the project, supervised data analysis, wrote, and corrected the manuscript. E.S. and C.P.F. provided funding.

## Conflicts of Interest

The authors declare no conflicts of interest.

## Supporting information


**Figure S1:** Comparison of gene expression profiles in adult hippocampal neuroblast populations across single‐cell RNA seq (scRNA‐seq) studies. Upset plot illustrating the overlap of expressed genes used to identify neuroblast populations between scRNA‐seq studies.

**Figure S2:** Age‐related changes in cell–cell communication within the neurogenic niche in the Wu et al. (2025) dataset. (A) CellChat analysis of young (3 months) and old (16–21 months) datasets from Wu et al. (2025) reveals a global reduction in both the number and strength of cellular interactions in the aged neurogenic niche. (B) Heatmap showing differential number of interactions or interaction strength among cells of young (3 months) and old (16–21 months) from Wu et al. (2025). The right‐colored bar plot displays the sum of the absolute values for each row, representing outgoing signaling. The bar height reflects the degree of change in the number of interactions or interaction strength between the two conditions. Red indicates an increase in signaling, while blue represents a decrease in the old dataset compared to young one. (C) Age‐associated alterations in signaling pathways in the neuroblast population of Wu et al. (2025).

## Data Availability

The code and data that support the findings of this study are openly available in aNSC_scRNAseq_comparison at GitHub (https://github.com/OPolzer/aNSC_scRNAseq_comparison). These data were derived from the following resources available in the public domain: Shin et al. (2015) (DOI: 10.1016/j.stem.2015.07.013); the accession number for the raw RNA sequencing data reported in this article is GEO: GSE71485. Artegiani et al. (2017) (DOI: 10.1016/j.celrep.2017.11.050); the accession number for the raw RNA sequencing data reported in this article is GEO: GSE106447. Batiuk et al. (2020) (DOI: 10.1038/s41467‐019‐14198‐8); the accession number for the raw RNA sequencing data reported in this article is GEO: GSE114000. Bielefeld et al. (2024) (DOI: 10.1038/s41467‐024‐49299‐6); the accession number for the raw RNA sequencing data reported in this article is GEO: GSE230942. Harris et al. (2021) (DOI: 10.1016/j.stem.2021.01.003); the accession number for the raw RNA sequencing data reported in this article is GEO: GSE159768. Hochgerner et al. (2018) (DOI: 10.1038/s41593‐017‐0056‐2); the accession number for the raw RNA sequencing data reported in this article is GEO: GSE9575. Wu et al. (2025) (DOI: 10.1038/s41593‐024‐01848‐4); the accession number for the raw RNA sequencing data reported in this article is GEO: GSE233363.
