# Supplementary material for: Cross‐Analysis of Single‐Cell Transcriptomic Datasets Reveals Conserved Neurogenic Gene Signatures and New Insights Into Neural Stem Cell Aging
Source: Aging Cell. 2025 Jun 4;24(8):e70106. doi: 10.1111/acel.70106 (PMC12341800; doi:10.1111/acel.70106)
Supplement: Supplementary file 1 — Figure S1: Comparison of gene expression profiles in adult hippocampal neuroblast populations across single‐cell RNA seq (scRNA‐seq) studies. Upset plot illustrating the overlap of expressed genes used to identify neuroblast populations between scRNA‐seq studies. [file ACEL-24-e70106-s002.pdf]

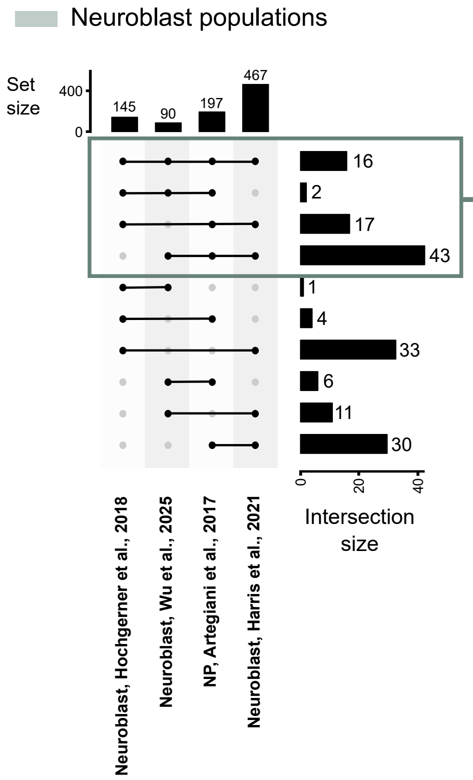

Gene overlap

among all clusters

Calb2 *Cd24a* *Elavl2* *Epha4*
Hist3h2ba *Igfbpl1* *Mex3b* *Mfap4*
Myt1l *Neurod1* *Sema3c* *Sox11*
Stmn1 *Stmn2* *Tac2* *Tubb3*

among three clusters

Auts2 *Bhlhe22* *Bzw2* *Cdk2ap1*
Cdk5r1 *Celf3* *Cnr1* *Crmp1*
Cttnbp2 *Dbn1* *Dcx* *Ddah2*
Dpysl3 *Dusp14* *Elavl3* *Elavl4*
Emx1 *Enc1* *Eomes* *Foxg1*
Hist3h2a *Igsf8* *Islr2* *Kif21a*
Kif5c *Lhx2* *Marcksl1* *Mex3a*
Mllt11 *Mpped1* *Mtss1* *Neurod2*
Neurod6 *Nfia* *Nfib* *Nfix*
Nhlh2 *Nnat* *Nr2f1* *Nrep*
Nsg2 *Pbx1* *Ppp1r14b* *Prdx2*
Ptprs *Rbfox2* *Rbfox3* *Rcor2*
Rnf165 *Slc17a6* *Sox4* *Srrm4*
Tbr1 *Tcf4* *Tmsb10* *Trim2*
Ttc28 *Tuba1a* *Tubb2b* *Tubb5*
Zbtb18 *Zbtb20*
