# Supplementary figures and images for: Cross‐Analysis of Single‐Cell Transcriptomic Datasets Reveals Conserved Neurogenic Gene Signatures and New Insights Into Neural Stem Cell Aging
Source: Aging Cell. 2025 Jun 4;24(8):e70106. doi: 10.1111/acel.70106 (PMC12341800; doi:10.1111/acel.70106)

**A**

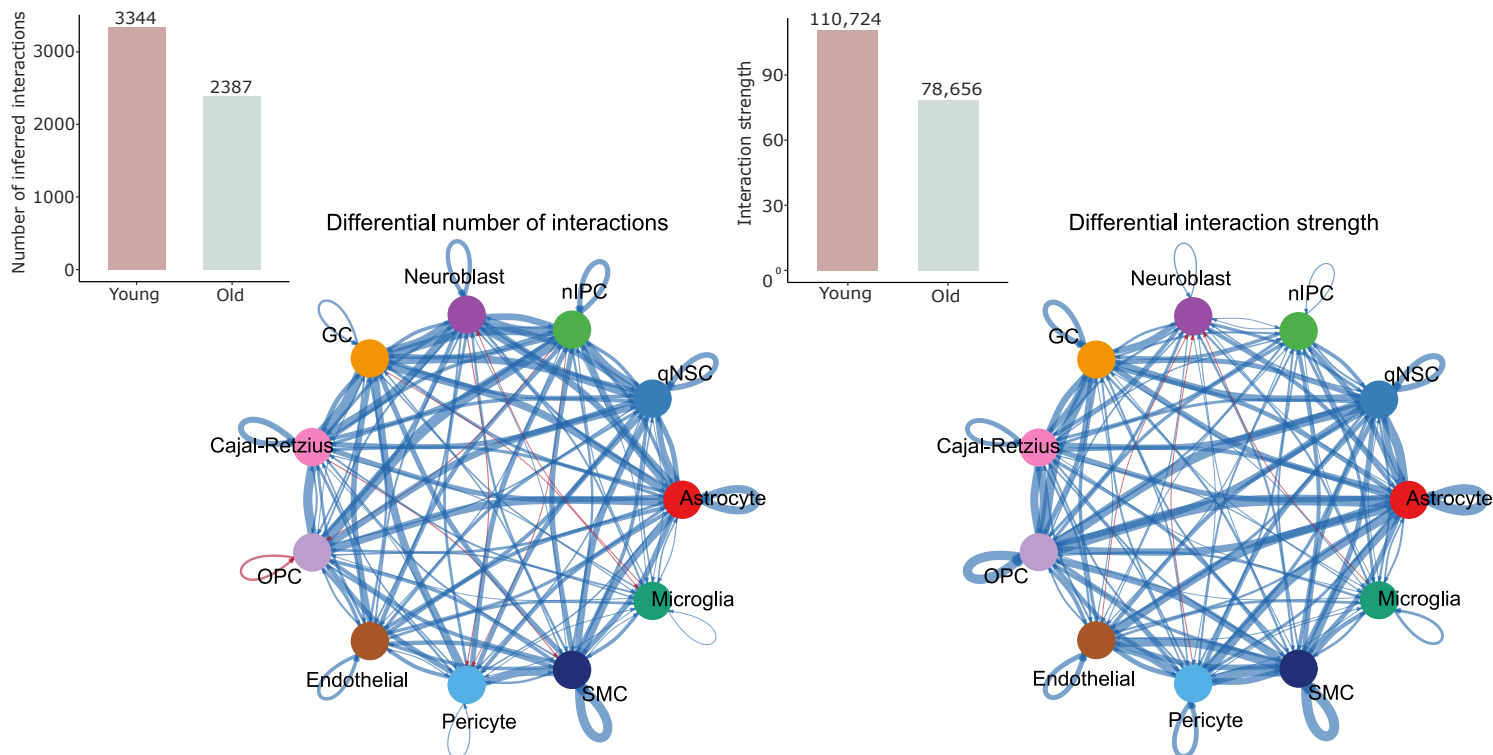

**B**

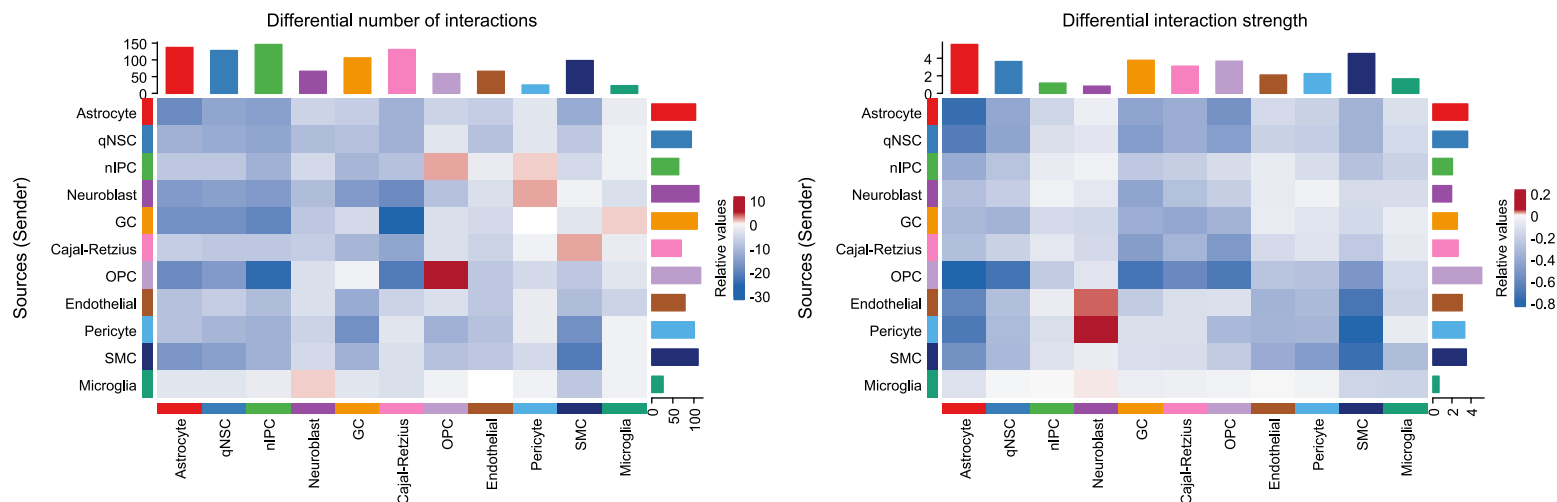

**C**

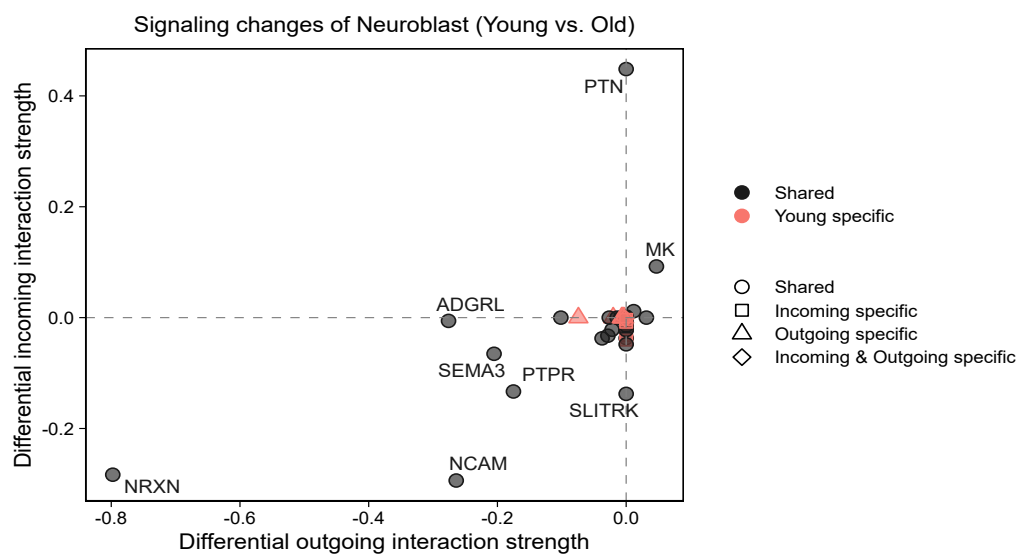

Supplement: Supplementary file 2 — Figure S2: Age‐related changes in cell–cell communication within the neurogenic niche in the Wu et al. (2025) dataset. (A) CellChat analysis of young (3 months) and old (16–21 months) datasets from Wu et al. (2025) reveals a global reduction in both the number and strength of cellular interactions in the aged neurogenic niche. (B) Heatmap showing differential number of interactions or interaction strength among cells of young (3 months) and old (16–21 months) from Wu et al. (2025). The right‐colored bar plot displays the sum of the absolute values for each row, representing outgoing signaling. The bar height reflects the degree of change in the number of interactions or interaction strength between the two conditions. Red indicates an increase in signaling, while blue represents a decrease in the old dataset compared to young one. (C) Age‐associated alterations in signaling pathways in the neuroblast population of Wu et al. (2025). [file ACEL-24-e70106-s001.pdf]
